# Shape Effect of Surface Defects on Nanohardness by Quasicontinuum Method

**DOI:** 10.3390/mi11100909

**Published:** 2020-09-30

**Authors:** Zhongli Zhang, Can Wang, Xiaowen Hu, Yushan Ni

**Affiliations:** 1 Department of Aeronautics and Astronautics, Fudan University, Shanghai 200433, China; zhangzl@simt.com.cn (Z.Z.); xwhu16@fudan.edu.cn (X.H.); 2 Shanghai Institute of Measurement and Testing Technology, Shanghai 201203, China; wangc@simt.com.cn

**Keywords:** nanoindentation, quasicontinuum method, surface defect, multiscale simulation, shape effect

## Abstract

Nanoindentation on a platinum thin film with surface defects in a rectangular shape and triangular shape was simulated using the quasicontinuum method to study the shape effect of surface defects on nanohardness. The results show that the nanohardness of thin film with triangular defects is basically larger than those with rectangular defects, which is closely related to the height of the surface defects at the boundary near to the indenter. Moreover, the triangular defect might have an enhancement effect on nanohardness by a certain size of the defects and the boundary orientation of the defect, where such an enhancement effect increases as the defect grows. Furthermore, the nanohardness decreases when the defect is folded from wide to narrow in the same atom cavity, and particularly expresses a more obvious drop when the height of the defects increases. In addition, larger sizes of the rectangular defect induce more reduction in nanohardness, while the nanohardness of the triangular surface defect is sensitive to the periodic arrangement of atoms changed by the boundary orientation of the defect, which is well explained and demonstrated by the calculation formula theory of necessary load for dislocation emission.

## 1. Introduction

Along with the development of the nanoindentation technique [1,2], which is a standard technique for evaluating the mechanical properties of thin films [3,4,5,6,7,8], an increasing number of scientists have focused on thin films with defects through simulations and experiments [9,10,11,12]. Reza Pejman et al. [13] investigated the effect of interfacial crack growth in the presence of a defect on deformation behavior and some mechanical properties such as hardness and the modulus of elasticity in a Cu/Si coating system. The results reveal that when the position of the defect is nearer to the shear stress concentration point, the crack will initiate sooner. Gabriel Plummer et al. [14] carried out the nanoindentation of the Ti_n+1_C_n_T_x_ MXenes via atomistic simulations, utilizing a parametrization of the ReaxFF interatomic potential, to understand the influence of point defects. Ni yushan et al. [15] has already investigated the comparison between surface defect situation and defect free situation by multi-scale simulation, and Zhang Zhongli et al. found the delay effect [16], the size effect [17], and the distance effect [18] of the surface defect through several nanoindentation simulations. Moreover, some scientists have begun to pay attention to the influence of the defect shape in material properties recently. Sultan Al-Owaisi et al. studied how the defect shapes and orientations affect the failure behavior of interacting defects through experiments [19]. Furthermore, in Micro-Electro-Mechanical Systems (MEMS) and the bulk micromachining field, surface defects are commonly seen in rectangular, polygonous and round shapes, which can be typically treated as the assembly of rectangular and triangular shapes when it is highly developed into the nano-scale. Therefore, it is of great significance to probe the shape effect on material properties, beginning from the analysis of surface defects in rectangular and triangular shapes, which actually have not been probed clearly yet. Our aim is to conduct analysis of the shape effect of surface defects in a rectangular shape and triangular shape on the nanohardness of Pt thin film by the quasicontinuum (QC) method [20], which can effectively conduct large scale simulation with low cost of calculating time and hardware requirement. We choose a platinum material, which has face-centered cubic (FCC) arrangement of the atoms in the crystal structure, just because the Pt group metals and in particular platinum alloys are indispensable in many fields of industrial application because of their outstanding physical and chemical properties [21].

## 2. Methodology

The quasicontinuum (QC) method is an effective mixed continuum and atomistic approach for simulating the mechanical response, especially in large-scale materials. The Ercolessi–Adams potential, which is one of the Embedded Atom Method (EAM) potentials [22,23,24,25], is used in this QC method, in order to describe the atomistic behavior. Strictly, it is crucial to state some necessary information about the convergence study of the model. In this model, “tolerance”, one of the factors in the QC method, is set as 10^−6^, which is used for solver and convergence checks. The command “conv” is set to check whether the convergence has been achieved within a loop, which will be terminated when no new elements have been generated since the previous convergence check. Moreover, it is necessary and significant to verify the validity of the QC method. In fact, some scientific workers have already paid attention to such aspect and finished assessing its reliability a few decades ago. Shenoy et al. of Brown University in the USA performed atomistic lattice statics using the quasicontinuum method and meanwhile carried out the analytic analysis within the Rice–Thomson and Peierls–Nabarro frameworks, and they found out the predications of the analytical models are qualitatively borne out by the atomistic simulations [26]. Knap and Ortiz have analyzed the accuracy and convergence characteristics of the quasicontinuum theory, where the effect of the summation rules on accuracy and the refinement tolerance have been assessed, and they have found that with sufficient mesh adaptivity, the quasicontinuum method is capable of simulating evolving microstructures comparable, in energetic terms, to those obtained from a full atomistic calculation [27].

The parameters for Pt in this potential are as shown in Table 1. In addition, it is necessary to state that the lattice constant (*a*_1_) of Pt is 0.392 nm, and the atomic lattice spacing in the [1 1 1] direction (d0) is 0.2263 nm.

**Figure 1:**
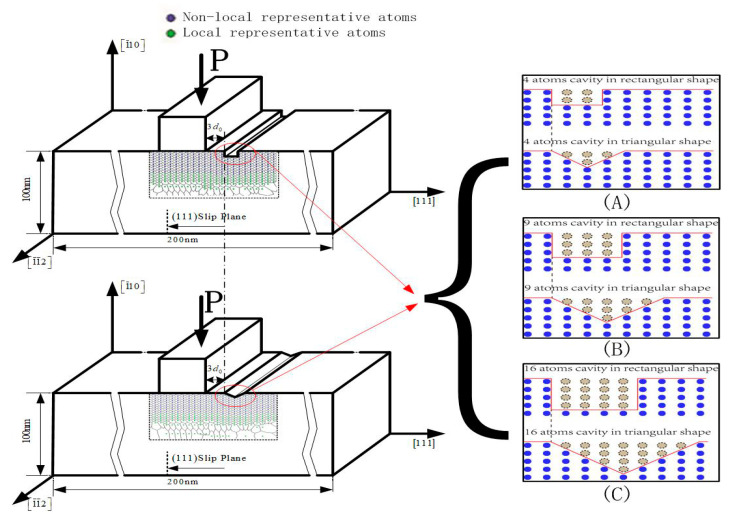
The schematic illustration of nanoindentation model with defects of a rectangular shape or triangular shape. (**A**) comparison model of defects with 4 atom cavity; (**B**) comparison model of defects with 9 atom cavity; (**C**) comparison model of defects with 16 atom cavity.

The schematic illustration of nanoindentation model with defects of a rectangular shape or triangular shape on the Pt thin film is as shown in Figure 1, where the width of the rigid rectangular indenter is 1.82 nm (eight times the lattice constant of Pt in the [1 1 1] direction (d_0_)). The substrate of thin Pt film (0.1 μm thick) is rigid and the symmetry boundary conditions on the sides of the model have been applied. Perfect stick conditions between the indenter and crystal are assumed that the friction and interactions between indenter tip atoms and film atoms are not considered. The number of atoms in contact with the indenter remains constant, such that they cannot slip out from under the indenter. Since the choice of the indenter tip shape is a critical point to the numerical nano-indentation results, it is necessary to claim the reason and make sufficient support for choosing the rectangular shaped indenter here. To be honest, based on the indentation simulations in recent years, a rectangular shaped indenter is not one of the most popular ones in the literature. However, it is undeniable that the rectangular shaped indenter is indeed one of the typical indenters, though a certain number of researchers preferred to use cylindrical or spherical indenters. Firstly, as well as the cylindrical-tipped or spherical-tipped indenters, the flat-tipped indenters have also been widely applied in the testing of material properties. Xiaofei Zhang et al. measured the fracture toughness of a poly methyl methacrylate (PMMA) plate using a newly developed flat-tip indentation method [30]. Heng Liu et al. calculated and presented the indentation moduli of transversely isotropic and layered half-spaces with imperfect interfaces using a flat-tipped rigid cylinder indenter [31]. Secondly, the rectangular indenter, which is typically one of the flat-tipped indenters mentioned above, has actually played an important role in nanoindentation. As early as 1995, the University of Nottingham in the U.K. performed analysis of the impression creep test method using a rectangular indenter for determining the creep properties in welds [32]. Shenoy et al. of Brown University in the USA examined the conditions for dislocation nucleation beneath a plane rectangular indenter using atomistic simulations [26]. Alizadeh et al. conducted two-dimensional quasicontinuum simulations of nanoindentation processes on aluminum thin films with nickel, copper, and silver coatings of different thicknesses under a rectangular indenter using the EAM potentials, and the obtained results were shown to be in good agreement with limited available experimental data and the analytical Rice–Thomson and Peierls–Nabarro dislocation models [33]. Furthermore, flat-tipped indenters or even rectangular shaped indenters have been commonly used in the punch field through simulations and experiments. Tadmor et al., who developed the QC method, particularly introduced the character of the rectangular indenter that is used in the punch indentation, where the number of atoms in contact with the indenter remains constant, such that they cannot slip out from under the indenter [34]. Bing-Feng Ju et al. conducted an experimental study of the elastic deformation as indented by a fine cylindrical, flat-ended punch in the presence of long-range intersurface forces [35]. Shiyun Shi et al. performed a series of experiments in order to examine the response of clamped steel plates loaded quasi-statically at their center by a rigid rectangular indenter, where the deformation modes of the tested plates are summarized based on the experimental observations and numerical simulations [36]. Based on the above discussion, the rectangular-shaped indenters do have particular significance in punch indentation. Due to its great advantages that the boundary of energy field (displacement field) remains unchanged when driven down into the $\left(\bar{1}10\right)$ surface, the rectangular-shaped indenter is of great necessity and importance to investigate the shape effect of defects near to the indenter. It can avoid severe damage of the defects’ shape by the contact area and guarantee the conclusion of the shape effect of defects not influenced by the distance factor between the pit and the indenter. The adjacent boundary between the defects and the indenter in this model is 3d_0_, which is chosen as being relatively moderate and proper, more sensitive to the shape effect based on our previous works [15,16] and some pre-simulations. The thickness of this model in the out-of-plane direction is 0.4801 nm, which is equal to the minimal repeat size applying the periodic boundary condition. To investigate the shape effect of surface defects, the surface defects of a rectangular shape and of a triangular shape with, respectively, 4, 9, and 16 atom cavity are taken into account.

For normal atomistic modeling standards, the dimensions of this simulation thin film are quite large, with approximately 1.3 million atoms or 4 million degrees of freedom (as shown, 0.1 μm in height and 0.2 μm in width in Figure 1). Though the molecular dynamic simulations with EAM potentials can already deal with more than 1 million atoms nowadays, the key problem is still the cost of calculating time and hardware requirement. Since personal computers are used in the majority of the simulation facilities, the QC method still has its advantages in saving calculating time within a PC environment, where the molecular dynamics model has been applied at the intense deformation region and a finite element model elsewhere in order to reduce the degrees of freedom without losing atomistic details. By comparison, there are only 4000 atoms or 12,000 degrees of freedom that have to be treated in this model, and can be easily finished in a few days using a personal computer.

## 3. Results and Discussion

**Figure 2:**
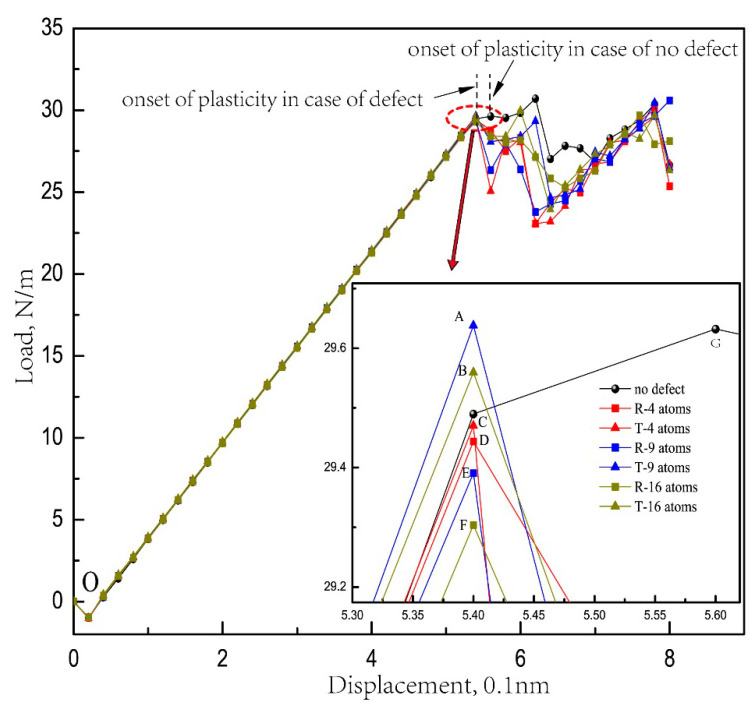
Load–displacement curves of nanoindentation on Pt thin film with and without defects.

The load–displacement curve obtained from nanoindentation simulations with its magnifying illustration which clearly shows the first load peak is presented in Figure 2, where load is expressed by length units of the indenter in the out-of-plane direction with its unit N/m, and “R” and “T” respectively represent the defects with a rectangular shape and triangular shape. It can be seen from Figure 2 that the load curves gradually increase until the first load peak during initial loading processes (OA-OG), which indicates the elastic stages of Pt thin film, where OA-OF is the case of surface defects and OG is carried out with no defect for comparison.

Take OG curve for instance—the load increases to a maximum value of 29.63 N/m when the load step reaches 0.56 nm at point A. Then, the load experiences continuous vibration and finally decreases to a minimum value of 27.04 N/m at 0.64 nm load depth. The hardness of Pt thin film with no defect is 16.28 GPa, according to the equation [37]: $H=\frac{P_{\max}}{A}$, where $P_{\max}$ is the maximum load and A is the contact area when indenting.

It can be found out from Figure 2 that the onset of plasticity of Pt thin film with defects has occurred early at load step 0.54 nm than that of nanoindentation with no defect at load step 0.56 nm, which is strongly related to the existence of defects, which is similar to published papers [15,16].

Further discussion can be made that among the indentations with surface defects (OA-OF), comparing the rectangular and triangular defects in the same atoms (the simulation models of (A), (B), (C) in Figure 1), the nanohardness of Pt thin film with triangular defects are basically larger than those with rectangular defects. It can might be well explained that in this simulation, the height of the rectangular defects at the boundary near to the indenter is larger than the one of the triangular defects, which induce more influence on nanohardness based on the previous research that the height effect of surface defect has more influence than the width on the yield load of a thin film [17]. In addition, a certain phenomenon should be noticed that the necessary load for elastic-to-plastic transition of triangular defect with a nine atom cavity is 29.638 N/m (point A in Figure 2), even larger than the one of nanoindentation on the defect-free surface 29.632 N/m (point G in Figure 2). Moreover, it can be easily found out from Figure 2 that the surface defect has an rectangular shape, whether with a 4, 9 and 16 atom cavity, the necessary load for elastic-to-plastic transition decreases respectively by 0.7%, 0.8%, and 1.1%, according to the value of nanoindentation on the defect-free surface, while the surface defect in triangular shape in a nine atom cavity is 0.02%, almost the same as the value of nanoindentation on the defect-free surface. Furthermore, the critical load in the triangular defects with a 16 atom cavity is also larger than that in the rectangular defect with a 4 atom cavity. Therefore, despite the uncertainty of the simulation data, which are extremely tiny, it can already be predicted based on the discussion above that the surface defect with a triangular shape might have an enhancement effect on nanohardness.

**Figure 3:**
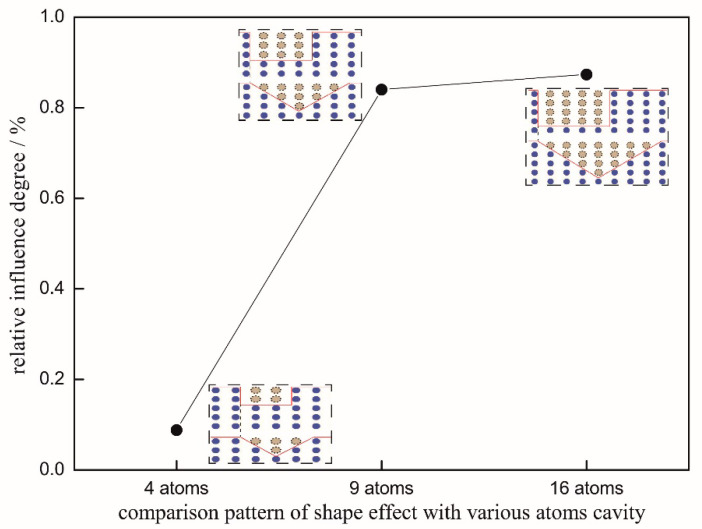
Relative influence degree on nanohardness between thin films with triangular defect and rectangular defect.

Furthermore, the relative influence degree η on nanohardness between thin films with triangular defect and rectangular defect with the same atom cavity can be calculated for further comparison by the equation: $\eta =\frac{N_{T}-N_{R}}{N_{R}}\times 100\%$, where $N_{T},N_{R}$ are, respectively, the nanohardness of Pt thin film with triangular defect and rectangular defect in the same atom cavity, namely a 4 atom cavity, 9 atom cavity, or 16 atom cavity in this simulation, just as Figure 3 shows. It can be easily figured out that the relative influence on nanohardness between the Pt thin films with triangular and rectangular defects in the same atom cavity gradually increases as the defects grows. Indeed, the differences in different defect shapes are generally less than 1%, as shown in Figure 3, as the relative influence degree seems tiny for the effect of the surface defect shape. So, it is necessary to make further discussion and explanation. Firstly, the difference is less than 1% in relative format but certainly large, around 160 MPa in absolute format (multiplied by the nanohardness of Pt, about 16 GPa). Secondly, along with the development of the bulk micromachining and nano-surface engineering, focusing on the various shapes of surface defects is increasingly popular and undeniably of great significance. In this paper, the micro-mechanism of how the different shapes of the surface defects influence the elastic–plastic transition of a thin film is more worthy of attention rather than the macro-aspects. Moreover, in consideration of the great significance of the study on the defect shape effect, even if there is no obvious effect between the rectangular defect and triangular defect, is also a kind of valid and significant conclusion.

**Figure 4:**
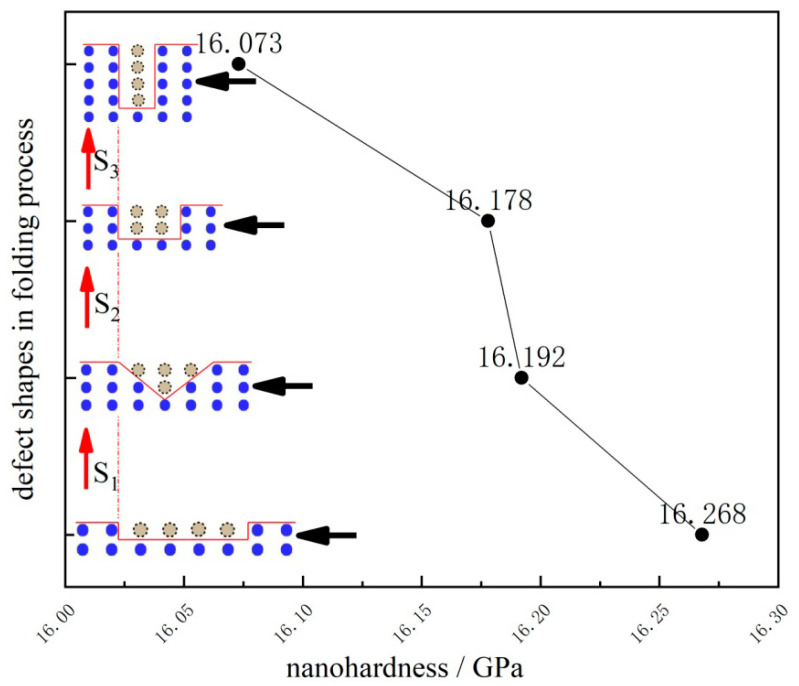
Nanohardness curve in defect folding process.

In order to make a more comprehensive investigation, study of the shape effect of defects in a four atom cavity during the folding process has also been carried out to observe how the necessary load for dislocation emission of Pt thin film changes. Four independent models have been simulated, as shown in Figure 4, where the surface defect in the four atom cavity is folded from wide to narrow, and the adjacent distance between the indenter and the defect is set as constant.

It is quite obvious that the nanohardness gradually decreases when the defect is folded from wide to narrow in the same atom cavity. The decline in the nanohardness in the three sections (S_1_, S_2_, and S_3_ in Figure 4) is of about 0.5%, 0.1% and 0.7%, respectively, suggesting that when the height of the surface defect increases (S_1_, S_3_ in Figure 4), it will induce a more obvious drop on nanohardness than the one with the defect height unchanged (S_2_ in Figure 4).

Moreover, comparing the Pt thin films with three rectangular defects of 4, 9 and 16 atom cavity (as shown rectangular symbols D, E, F in Figure 2), it can be easily figured out from Figure 2 that the larger sizes of the rectangular defect induce a greater reduction in nanohardness. This is consistent with the size effect of surface rectangular defects of FCC thin film when indenting in the $\left[\bar{1}10\right]$ direction [17,38].

**Figure 5:**
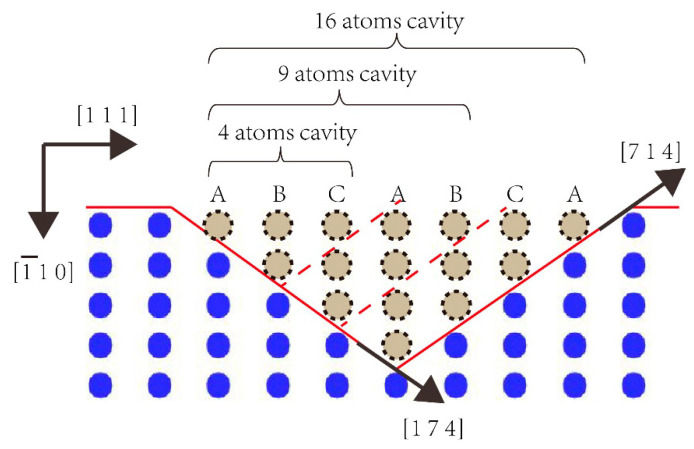
The boundary orientation sketch of the triangular defects.

However, it is more complicated in the nanohardness of Pt thin films with triangular defects (as shown triangular symbols A, B, C in Figure 2), where Pt thin film with triangular defects with a nine atom cavity has the biggest nanohardness, while the one with triangular defect with a four atom cavity has the lowest value. According to the crystal structure of Pt, it can be calculated that the two orientations of the triangular defect are, respectively, [1 7 4] and [7 1 4], as shown in Figure 5, where the direction we are looking at the schematics is $\left[\bar{1}\bar{1}2\right]$. It is necessary to state here that the model in this paper is in fact two-dimensional, where the thickness of this model in the $\left[\bar{1}\bar{1}2\right]$ direction is only the thickness of the unit cell, which equals the minimal repeat size, though it seems to be three-dimensional.

First of all, the boundary orientation of the triangular defect might reasonably explain the enhancement effect induced by the triangular defect, that since $\left(\bar{1}10\right)$ surface is the atomic close-packed plane, while the (1 7 4) surface is not, there would be relatively more lattice resistance caused by the [1 7 4] boundary of the triangular defect when the dislocation glides deep into the $\left(\bar{1}10\right)$ surface. Furthermore, it has been well recognized that there is a periodic arrangement of atoms as “ABCABC” on {111} atomic close-packed planes in face-centered cubic metal [39]. Such a phenomenon among the triangular defects of 4, 9 and 16 atom cavities can be likely due to the periodic arrangement of atoms changed by the cutoff of [1 7 4] and [7 1 4]. It might be predicted that the cutoff “C/A by [1 7 4] and C/A by [7 1 4]” in the 4 atom cavity is relatively the most close-packed that facilitate sliding among the three triangular defects in this simulation, while the cutoff “C/A by [1 7 4] and B/C by [7 1 4]” in a 9 atom cavity is the least close-packed and the cutoff “C/A by [1 7 4] and A/B by [7 1 4]” in a 16 atom cavity is the medium. That might make a reasonable explanation why the Pt thin film with the triangular defect with a nine atom cavity has the biggest nanohardness, and the one with the triangular defect with a four atom cavity has the lowest.

**Figure 6:**
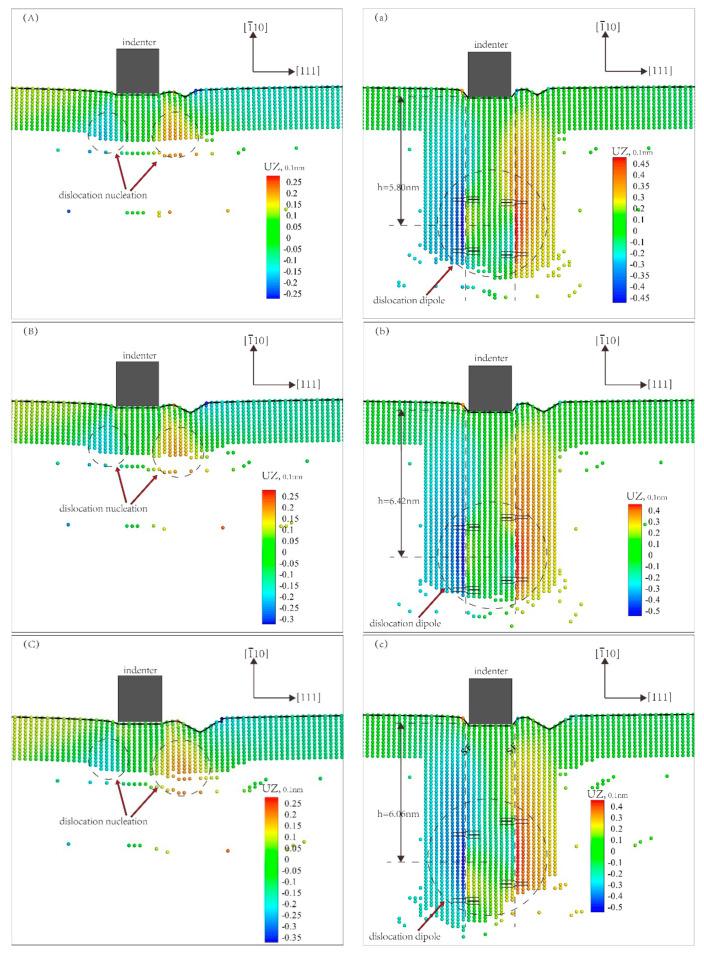
Snapshot of atoms under indenter and corresponding out-of-plane displacement plot, where UZ is atom displacement at out-of-plane: (**A**) 4 atom cavity at point C in Figure 2 (dislocation nucleation); (**a**) 4 atom cavity at load step 0.62 nm (dislocation emission); (**B**) 9 atom cavity at point A in Figure 2 (dislocation nucleation); (**b**) 9 atom cavity at load step 0.64 nm (dislocation emission); (**C**) 16 atom cavity at point B in Figure 2 (dislocation nucleation); (**c**) 16 atom cavity at load step 0.64 nm (dislocation emission).

In order to make a further study on Pt thin films with triangular surface defects of 4, 9 and 16 atom cavities, the atomic positions and out-of-plane displacements have been probed. Figure 6 shows the snapshot of atom structure both at the critical load step of elastic-to-plastic transition and at the step of dislocation emission, where dimensions and displacements are within 0.1 nm. The nucleated dislocations can be easily seen beneath the indenter, as displayed in Figure 6A–C. Two Shockley partial dislocations that bound a stacking fault have emitted through dislocation glide deep into the $\left(\bar{1}10\right)$ plane with its dipole depth being 5.80, 6.42 and 6.06 nm, corresponding the simulation models of Pt thin films with triangular surface defects with 4, 9 and 16 atom cavities, as shown in Figure 6a–c, where the Burgers vector ($\vec{b}$) is 0.277 nm.

The critical load for dislocation emission is discussed and carried out according to the calculation formula of necessary load for elastic-to-plastic transition that applied by Tadmor [34] as follows:

(1) $$P_{cr}=\frac{\mu b}{4\pi (1-v)}\ln\frac{32h(h+2a)a^{2}}{b^{4}}+2\gamma_{111}+\frac{1}{2}kb$$

where *P_cr_* is the critical load value for elastic-to-plastic transition, μ is the shear modulus of Pt crystal, *k* is the slope of elastic stage in the load-displacement curve, *h* is the depth of emitted dislocation dipole, *a* is the half width of indenter, γ_111_ is the energy of (111) surface of Pt crystal. The value of the shear modulus μ can be computed effectively from ***C***_11_, ***C***_12_, and ***C***_44_ by performing a Voigt average leading to the relations as following [34]:

(2) $$\mu =\frac{1}{5}\left(C_{11}-C_{12}+3C_{44}\right)$$

which gives μ 66 GPa of Pt crystal through the parameters of EAM potential in Table 1.

**Figure 7:**
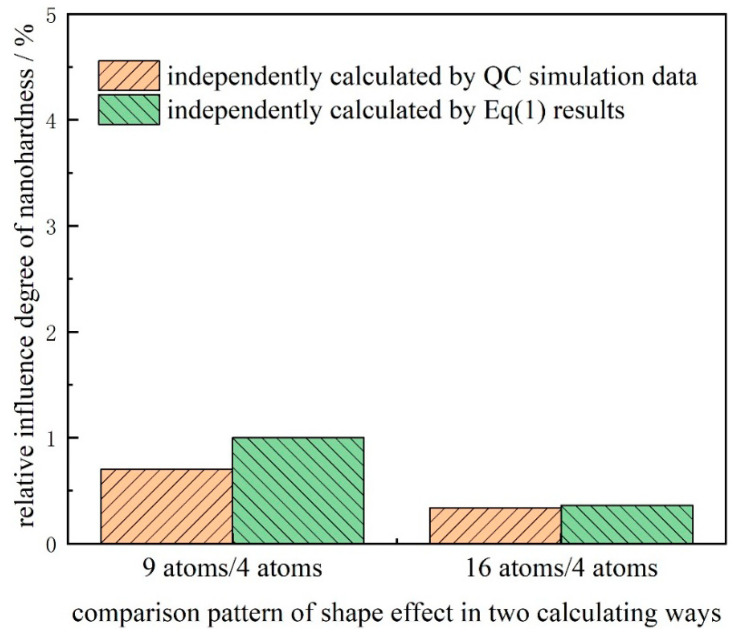
Relative influence on the nanohardness of triangular surface defects in two calculation methods.

Figure 7 shows the relative influence on nanohardness of triangular surface defects independently calculated by QC simulation data or the results from Equation (1). It is necessary to further declare that the yellow symbol is the data by the equation: ${\eta_{\mathrm{QC}}}^{\prime }=\frac{N^{\mathrm{QC}}{}_{\mathrm{Ti}}-N^{\mathrm{QC}}{}_{T4}}{N^{\mathrm{QC}}{}_{T4}}\times 100\%$, where $N^{\mathrm{QC}}{}_{\mathrm{Ti}}$ is the nanohardness of triangular defect with a 9 atom cavity or 16 atom cavity, and $N^{\mathrm{QC}}{}_{T4}$ is the nanohardness of triangular defect with a 4 atom cavity, where the nanohardness is obtained through the load-displacement curve in QC simulation, as shown in Figure 2. The green symbol is the data by the equation: ${\eta_{\mathrm{eq}}}^{\prime }=\frac{N^{\mathrm{eq}}{}_{\mathrm{Ti}}-N^{\mathrm{eq}}{}_{T4}}{N^{\mathrm{eq}}{}_{T4}}\times 100\%$, where $N^{\mathrm{eq}}{}_{\mathrm{Ti}}$ is the nanohardness of triangular defect with a 9 atom cavity or 16 atom cavity, and $N^{\mathrm{QC}}{}_{T4}$ is the nanohardness of triangular defect with a 4 atom cavity, where the nanohardness is calculated by the formula (Equation (1)), applying each depth of emitted dislocation dipole *h*, as shown in Figure 6.

This shows that the data from QC simulation and formula calculation are pretty close, and more significantly in a same trend where the triangular defect with a 9 atom cavity has a greater influence on nanohardness than the triangular defect with a 16 atom cavity. That is to say, the complicated phenomenon shown in Figure 2 among the triangular defects with three sizes, where the triangular defect with a 9 atom cavity has the biggest nanohardness, has been explained and demonstrated to some extent by the calculation formula of necessary load for dislocation emission, due to the emission depth of dislocation dipole of two Shockley partial dislocations from the micro perspective.

## 4. Conclusions

The shape effect of surface defects on nanohardness has been probed though the QC method in this paper. Several nanoindentation models of Pt thin films with surface defects with a rectangular shape and triangular shape have been taken into account, and such conclusions can be drawn as follows:

(1) The nanohardness with triangular defects is basically larger than those with rectangular defects, which is closely related to the height of the surface defects at the boundary near to the indenter.
(2) The triangular surface defect might have an enhancement effect on nanohardness due to the certain size of the defects and the boundary orientation of the defect, where such an enhancement effect increases as the defects grows.
(3) The nanohardness decreases when the defect is folded from wide to narrow in the same atom cavity, and particularly expresses a more obvious drop when the height of the defects increases.
(4) The larger sizes of the rectangular defect induce more reduction in nanohardness, while the nanohardness of the triangular surface defect is sensitive to the periodic arrangement of atoms changed by the boundary orientation of the defect, which has been explained and demonstrated to some extent by the calculation formula theory of necessary load for dislocation emission.

## Figures and Tables

**Table 1:** The parameters for Pt in EAM potential and experiment.

Content	EAM	Experiment
Poisson (*ν*)	0.39	0.39
(1 1 1) surface energy (γ_111_)	1.46 J/m^2^	1.47 J/m^2^ [a]
***C***_11_	302 GPa	346.7 GPa [b]
***C***_12_	206 GPa	250.7 GPa [b]
***C***_44_	78 GPa	76.5 GPa [b]

[a] The experimental value of (1 1 1) surface energy γ_111_ is 1.47 J/m^2^ [28]. [b] The experimental values extrapolated to ***T*** = 300 K [29].

## Data Availability

The research data required to reproduce this work reported in this manuscript is free to obtain by email.
